# Did we find a copycat? Do as I Do in a domestic cat (*Felis catus*)

**DOI:** 10.1007/s10071-020-01428-6

**Published:** 2020-09-18

**Authors:** Claudia Fugazza, Andrea Sommese, Ákos Pogány, Ádám Miklósi

**Affiliations:** 1 Department of Ethology, Eötvös Loránd University, Budapest, Hungary; 2 MTA-ELTE Comparative Ethology Research Group, Budapest, Hungary

**Keywords:** Social learning, Do as I do, Cat, Two-action method, Imitation, Response facilitation

## Abstract

This study shows evidence of a domestic cat (*Felis catus*) being able to successfully learn to reproduce human-demonstrated actions based on the Do as I Do paradigm. The subject was trained to reproduce a small set of familiar actions on command “Do it!” before the study began. To test feature–contingent behavioural similarity and control for stimulus enhancement, our test consisted of a modified version of the two-action procedure, combined with the Do as I Do paradigm. Instead of showing two different actions on an object to different subjects, we applied a within-subject design and showed the two actions to the same subject in separate trials. We show evidence that a well-socialized companion cat was able to reproduce actions demonstrated by a human model by reproducing two different actions that were demonstrated on the same object. Our experiment provides the first evidence that the Do as I Do paradigm can be applied to cats, suggesting that the ability to recognize behavioural similarity may fall within the range of the socio-cognitive skills of this species. The ability of reproducing the actions of a heterospecific human model in well-socialized cats may pave the way for future studies addressing cats’ imitative skills.

**Electronic supplementary material:**

The online version of this article (10.1007/s10071-020-01428-6) contains supplementary material, which is available to authorized users.

## Introduction

Despite an increased research interest in understanding cat behaviour and cognition (e.g., Pongrácz et al. 2019; Vitale Shreve et al., 2017a, 2015, very little is known )about various aspects of their socio-cognitive capacities. Particularly, while social learning and imitation have been in the focus of research in many other species (e.g., orangutans, Call 2001; rats, Heyes et al. 1994; dogs, Topál et al., 2006), we have very limited and context-specific data on cats’ social learning skills. The extant knowledge about social learning in cats typically involves learning how to obtain food from a conspecific demonstrator. Some studies showed that cats tend to pay attention to conspecifics when food is involved (Adler 1955; Winslow 1944). Kittens were shown to be more likely to press a lever to obtain food if they observed their mother doing so. To some extent, this happened also if they observed an unfamiliar conspecific (Chesler 1969). In the latter study, a control group of kittens that did not observe the demonstration that was included to control for individual learning. However, the exact process of social learning cannot be established, because social facilitation and various social learning processes may have contributed to better performance in experimental as opposed to control kittens. Adult cats also seem to benefit from observing an experienced conspecific (Herbert and Harsh 1944; John et al. 1969). Adler (1955) used the so-called Warden Duplicate Cage (Warden et al. 1935) to test social learning in a problem-solving situation in cats. The demonstrator animal manipulated an object to obtain food and the performance of observer cats was measured to investigate whether they benefitted from the demonstration in terms of shorter time needed to solve the task. The subjects were divided in different experimental groups exposed to different variations of the demonstration. Similar to other early attempts to investigate social learning, this study is also lacking the specific control conditions to separate imitation, emulation and stimulus enhancement. This does not allow drawing conclusions on the process involved. We suggest that the observer cats’ response can most likely be explained by stimulus enhancement (Zentall 2006). In fact, it is not possible to exclude that the observers’ attention was drawn to the object that was manipulated by the demonstrator and that this, as a consequence, increased the probability of acting on that object. The authors also observed that, while the observer cats tended to benefit from the demonstration in previous trials (i.e., when the authors argued social learning was more likely to play a role), there was a big variation in the cats’ responses in subsequent trials, when individual learning of how to solve the task efficiently was also likely to occur. They concluded that “*the finding that this advantage is not very permanent, and that individual differences in trial-and-error learning tend to show up strongly in subsequent trials, points to the fact that cats do not make much use of observation learning*” (Adler 1955).

Despite the absence of recent and more controlled studies on social learning and imitation in cats, there is growing interest in the socio-cognitive skills of this species. For example, cats were reported to rely on human gestural signals (Miklósi et al. 2005) and gaze direction (Pongrácz et al. 2019) in addition to engaging in social referencing with humans, in presence of a potentially frightening novel stimulus (Merola et al. 2015). As a result of domestication, cats share their natural environment with humans (Bradshaw et al. 1999). Cats living in human families often have more exposure to humans than to conspecifics and they predominantly interact with humans in various activities (play, feeding, etc.) from an early age. Evolution and development in the human social environment place cats among the few especially interesting domesticated species in which there is ample possibility for social learning from humans. Particularly, testing cats’ ability to reproduce body movements demonstrated by a heterospecific model constitutes an interesting case, because it provides insights into how the observer may represent the actions of the heterospecific model (e.g., whether and how it represents and maps the model’s different body parts as related to its own).

In this study, we focus on feature–contingent behavioural similarity between the observed and the replicated behaviour in cats. Importantly, the selection of matching behaviour may depend on the ability to recognize behavioural similarity, and it is this ability on which our study focuses.

Several other perceptual factors may increase the attention of the observer towards the object manipulated by the demonstrator or his goal and, consequently, increase the probability of a similar behavioural response by chance—e.g. stimulus enhancement, (Thorpe 1963) and goal emulation (Tomasello 1990). Importantly, in these cases, behavioural similarity does not rely on the recognition of the body movement of the demonstrator. Thus, the exclusion of these processes is necessary to confirm that similarity between the behaviour of the demonstrator and that of the observer relies on the ability to execute a specific form of a motor action after observation (Zentall 2006).

Depending on the definition used by different authors, the execution of a specific motor action after its observation is called imitation (e.g. Whiten and Ham 1992) or response facilitation (Byrne 1994; Hoppit et al. 2007). The latter involves detection and encoding of a perceived action, and selection and control of an already known motor response, so that there is clear similarity between the observed action (as perceptual input) and the motor response. The choice seems to depend on the novelty or probability of the observed and reproduced behaviour (Thorpe 1963). However, especially in species with a very flexible and wide-ranging behavioural repertoire, like cats, measuring how probable an action is for a given individual can be challenging, making this definition difficult to use from a methodological point of view. Moreover, novelty is a relative concept that may refer to different aspects of behaviour, such as shape, orientation or extent, and the action might have been performed before, but in a different context. (Whiten and Custance 1996; Whiten 1998).

We aimed at testing the ability to reproduce actions demonstrated by a heterospecific model in a cat that was trained to match her behaviour to a small set of familiar (i.e., already trained) human-demonstrated actions with the Do as I Do method (Topál et al. 2006; Fugazza and Miklósi 2014) by her owner, before the study began. To control for alternative processes that may enhance the probability of a similar response, we combined the Do as I Do paradigm with the two-action procedure (Akins and Zentall 1996; Van De Waal et al. 2012; Dawson and Foss 1965). A similar method was already successfully applied in dogs (e.g., Fugazza and Miklósi 2014; Fugazza et al. 2016). According to the two-action method, two different actions are shown on the same object. This allows excluding stimulus-enhancement and goal emulation because, if the behavioural similarity relies on those processes, then the observer is expected to match the object used by the demonstrator but not the action, whereas if the observer is able to identify the action of the demonstrator, then he is expected to match its body movements.

We applied a modified version of the two-action method, in which, instead of testing different subjects upon reproducing two different actions on the same object, we tested the same subject on two different actions (on different occasions) on the same object (for a similar procedure on dogs see Fugazza and Miklósi 2014).

## Materials and methods

### Subject and preliminary training

Our subject was an 11-year-old female cat, called Ebisu, living with her owner in Ichinomia (Japan). The owner, Fumi Higaki, is a professional dog trainer, experienced with the use of the Do as I Do method to train dogs. She reported that the cat had always been exceptionally motivated for food, a condition that allowed her to train the cat relatively easily by applying the same operant conditioning-based training methods commonly used for dogs. Ebisu was trained by her owner with the Do as I Do protocol to match her behaviour to actions that were demonstrated by a human (Topál et al. 2006; Fugazza and Miklósi, 2014). The Do as I Do training took place in the pet shop of the owner, where the cat lived, between May and September 2019. The training protocol is based on Topál et al. (2006) and Fugazza and Miklósi (2014) and it involves two steps. First, the subject is trained by operant conditioning techniques to match her behaviour to three familiar actions (i.e. actions that the cat was previously trained to perform on verbal cue) demonstrated by her owner, on command “Do it!”. Second, this command is generalized to three other familiar actions, after which the “Do it!” command can be used as a rule for the subject to reproduce novel actions presented by the demonstrator.

The actions that the owner trained and taught to the cat to imitate during the Do as I Do training included: spin, stand up on the hind legs, touch a wobbling toy with a paw, open a little plastic drawer, bite a rubber string and lay down (Table 1). The training included overall 21 training sessions of 3–10 trials each (Table 2). After the 21st training session, the owner taught the cat two new actions using the “action matching rule” (i.e., demonstrating the action and giving the “Do it!” command): open a sliding lid and climb with forearms on a book. When the owner demonstrated opening a sliding lid—i.e. sliding to a side the lid of a stainless-steel container—Ebisu successfully slid the lid to a side on the first trial. When the owner demonstrated climbing with forearms on a book, at first, the cat touched it with one paw without moving from her sitting position. In the next 4 similar demonstrations, the cat did the same; therefore, the owner placed the book further from the cat (at approximately 30 cm from her). On the 1st occasion with this new set-up, Ebisu placed her forearms on the book, thereby matching the demonstration (Table 1).

**Table 1:** Description of the demonstrations and the cat’s behaviours considered as matching during Do as I Do training and when teaching novel actions prior to the test

Action	Demonstration	Cat action
Spin	The owner turns her body around her vertical axis	The cat turns her body around her vertical axis
Stand up on hind legs	The owner stands on her tows and raises her arms	The cat stands on her hind legs and raises her front paws
Touch wobbling toy with paw	The owner touches a wobbling toy with her hand	The cat touches a wobbling toy with her front paw
Pull drawer open	The owner pulls a little plastic drawer open using her hand	The cat pulls a little plastic drawer open using her front paw
Bit rubber string	The owner takes in her mouth a rubber string that is hanging from a hanger	The cat takes in her mouth a rubber string that is hanging from a hanger
Lay down	The owner lays her body horizontally on the floor	The cat lays her body horizontally on the table
Climb with front paws on a book	The owner puts her forearms on a book	The cat places her forearms on a book, as if laying with her front body part on it
Open a sliding lid	The owner uses her hand to slide to a side and open the plastic lid of a small container	The cat uses her front paw to slide to a side and open the plastic lid of a small container

The actions “climb with front paws on a book” and “open a sliding lid” were not previously trained. All other described actions were previously trained by the owner

**Table 2:** Do as I Do training of the cat

Training session	Date	Trial	Demonstration	Action matching	Notes
1	20 May	1	Stand up on hind legs	1	Prompted by trained cue
		2	Spin	0	
		3	Spin	1	Prompted by trained cue
		4	Touch wobbling toy with paw	1	Prompted by trained cue
		5	Spin	1	
		6	Spin	1	Prompted by trained cue
		7	Touch wobbling toy with paw	1	Prompted by trained cue
		8	Stand up on hind legs	1	Prompted by trained cue
2	24 May	1	Spin	1	Prompted by trained cue
		2	Touch wobbling toy with paw	0	
		3	Touch wobbling toy with paw	0	
		4	Stand up on hind legs	1	Prompted by trained cue
		5	Touch wobbling toy with paw	0	
		6	Touch wobbling toy with paw	1	Prompted by trained cue
		7	Touch wobbling toy with paw	1	Prompted by trained cue
3	03 June	1	Spin	1	Prompted by trained cue
		2	Stand up on hind legs	1	Prompted by trained cue
		3	Touch wobbling toy with paw	1	Prompted by trained cue
4	15 June	1	Spin	0	Did not look at demonstration
		2	Spin	0	Did not look at demonstration
		3	Touch wobbling toy with paw	1	
		4	Stand up on hind legs	0	Did not look at demonstration
		5	Stand up on hind legs	1	Prompted by trained cue
		6	Spin	1	Prompted by trained cue
5	21 June	1	Touch wobbling toy with paw	1	
		2	Stand up on hind legs	1	Prompted by trained cue
		3	Touch wobbling toy with paw	1	
		4	Spin	0	
		5	Spin	0	
		6	Touch wobbling toy with paw	1	
6	22 June		No data		
7	07 July	1	Touch wobbling toy with paw	1	
		2	Spin	1	Prompted by trained cue
		3	Stand up on hind legs	1	
		4	Spin	1	
8	13 July	1	Spin	1	
		2	Touch wobbling toy with paw	1	
		3	Stand up on hind legs	0	
		4	Stand up on hind legs	1	
9	19 July	1	Spin	1	
		2	Touch wobbling toy with paw	1	
		3	Stand up on hind legs	1	
10	19 July	1	Stand up on hind legs	1	
		2	Touch wobbling toy with paw	1	
		3	Spin	1	
11	28 July	1	Touch wobbling toy with paw	0	Did not look at demonstration
		2	Touch wobbling toy with paw	1	
		3	Stand up on hind legs	1	
		4	Pull drawer open	1	
		5	Bite	0	Did not look at demonstration
		6	Bite	0	
		7	Spin	0	
		8	Bite	0	
		9	Bite	0	
		10	Pull drawer open	1	
12	05 August	1	Pull drawer open	1	
		2	Down	1	Prompted by trained cue
		3	Stand up on hind legs	1	
		4	Bite	0	
		5	Bite	0	
		6	Touch wobbling toy with paw	1	
		7	Spin	0	
		8	Stand up on hind legs	0	
		9	Stand up on hind legs	1	
13	05 August	1	Down	0	
		2	Down	0	
		3	Spin	0	
		4	Bite	1	
		5	Stand up on hind legs	0	
		3	Pull drawer open	1	
14	08 August	1	Stand up on hind legs	1	
		2	Touch wobbling toy with paw	1	
		3	Pull drawer open	1	
		4	Spin	1	Prompted by trained cue
		5	Spin	1	
		6	Bite	1	
		7	Down	0	Did not look at demonstration
		8	Down	0	Did not look at demonstration
15	08 August	1	Spin	1	
		2	Bite	1	
		3	Down	0	Did not look at demonstration
		4	Down	0	Did not look at demonstration
		5	Pull drawer open	1	
16	23 August	1	Bite	1	
		2	Stand up on hind legs	0	
		3	Stand up on hind legs	1	
		4	Open	1	
		5	Spin	0	
17	23 August	1	Down	1	Prompted by trained cue
		2	Down	0	
		3	Spin	0	
		4	Bite	0	Did not look at demonstration
		5	Down	0	Did not look at demonstration
		6	Pull drawer open	1	
		7	Spin	0	
18	29 August	1	Bite	0	Did not look at demonstration
		2	Pull drawer open	1	
		3	Down	1	
		4	Touch wobbling toy with paw	1	
		5	Spin	0	
		6	Stand up on hind legs	1	
		7	Bite	0	Did not look at demonstration
		8	Bite	0	
20	29 August	1	Spin	1	Prompted by trained cue
		2	Bite	0	
		3	Bite	1	
		4	Spin	0	Did not look at demonstration
		5	Spin	0	
21	07 September	1	Spin	1	
		2	Bite	0	
		3	Bite	1	
		4	Down	1	
		5	Pull drawer open	1	
		6	Touch wobbling toy with paw	1	
		7	Stand up on hind legs	1	

The table reports the number of training sessions, the number of trials within each training session, the action demonstrated in each trial, the action matching performance of the cat (1 = matched the demonstration; 0 = did not match the demonstration) and, in the column named “notes” we report whether the cat’s performance was prompted by a trained verbal cue. Additionally, trials in which the cat did not look at the demonstration are noted

In March 2019, the cat was diagnosed with renal disease (stage 3), but the owner reported that her motivation for food and her physical condition had been apparently good and stable until September 2019, when the cat started to show a decreased appetite and a lower level of activity.

### Experimental procedure

**Fig. 1:**
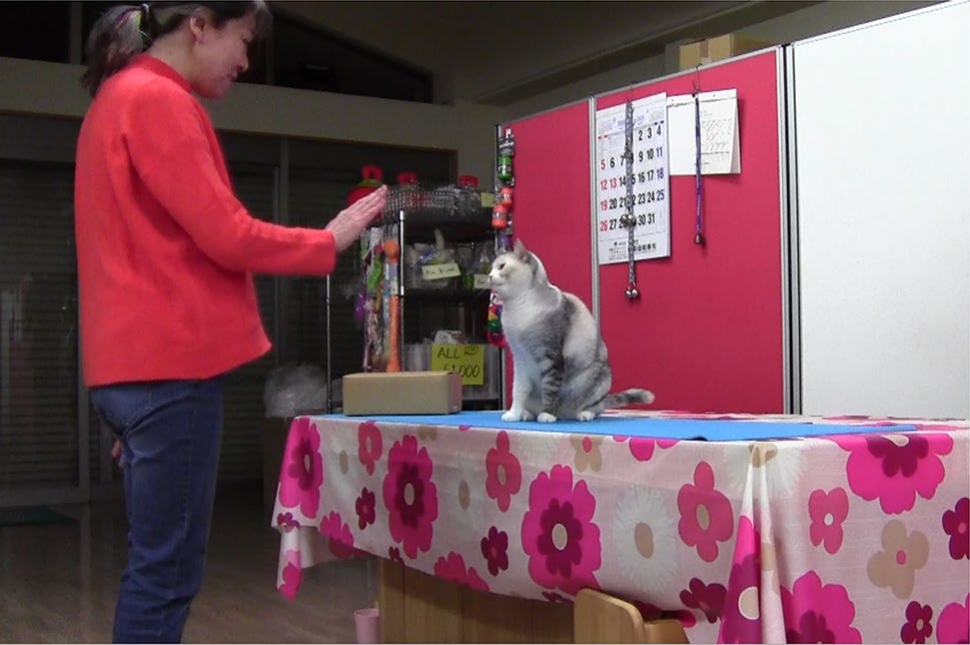
Test set-up. The owner is facing the cat, which is on the table, at 50 cm from the cardboard box used in the tests

The tests were conducted in December 2019 in the pet shop where the training took place, in the evenings, when the shop was closed to the public. In each test, a cardboard box (27 × 18 × 10 cm) was displayed on a table (180 × 90 × 72 cm, the same table that had also been used during training). The owner was standing in front of the table and provided cues to induce the cat to go on it and to sit in front of her. The owner and the cat were facing each other, and the box was placed laterally at 50 cm from the cat’s position (Fig. 1).

At the beginning of each trial, the owner attracted the cat’s attention using food, petting and vocalizations and induced the cat to sit and stay in front of her using signals known by the cat. As soon as the owner noticed that the cat was looking at her (which could be further encouraged by vocalizations), the owner performed the demonstration, then returned to her starting position facing the cat and gave the “Do it!” command. The cat’s behaviour was then observed for maximum 20 s (or until the cat performed an action). To avoid any possible inadvertent cueing on the part of the owner, the owner looked straight ahead while giving the “Do it!” command (Fugazza and Miklósi, 2014). We also note that the two-action method intrinsically controls for cues that may direct the animal to a given direction or object because, even a directional cue inviting the subject to move towards the object would not provide information as to what action to perform on it.

Following the two-action procedure (Akins and Zentall 1996), two actions of similar difficulty (A and B) were defined in advance but, while in previous work where the two-action method was applied, only one of these actions was ever demonstrated to a given subject (between-subject design), we demonstrated both actions—in different test sessions—to our subject (within-subject design).

**Fig. 2:**
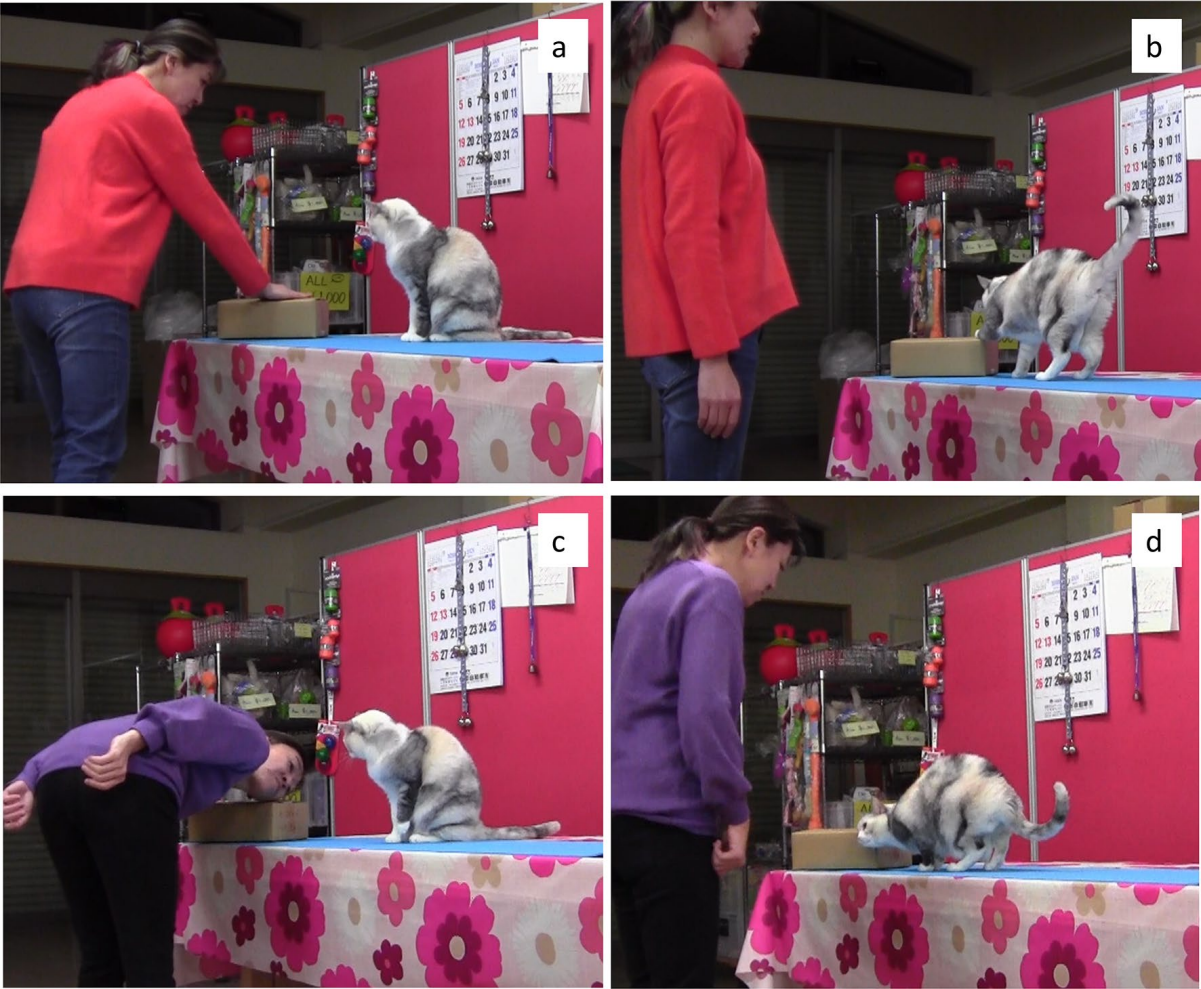
Illustrations of the two demonstrated actions (1a and 1c) and of the cat’s performance after the “Do it!” command (1b and 1d). 1a: Demonstration of action A: the owner raises her right hand and touches the box with it; 1b: cat’s action scored as matching the demonstration of action A; 1c: Demonstration of action B: the owner bends down to rub her face on the box; 1d: Cat’s action scored as matching the demonstration of action B

We used the two following actions on the box: Action A: the owner raised her right hand and touched the box with it; Action B: the owner bent down to rub her face on the box (Fig. 2 and video S1). The two object-related actions (A and B) were chosen to represent tasks of similar difficulty (simple interactions with objects), and similar performance between the two actions confirmed this (see Results). While touching an object with paw was a trained action (but the object on which the demonstration was done was novel), rubbing the face against something had never been trained.

Due to her disease, Ebisu’s motivation for food had reduced significantly in the period when we conducted the test (December 2019). This forced us to expose her to a limited amount of trials per test occasion and to a limited number of test occasions as well. Every test session consisted of 3 trials in which the same action (either A or B) was demonstrated, every time followed by the “Do it!” command. We planned to run 6 test sessions of 3 trials each, one test session per day with an inter-test interval of minimum 1 day, maximum 2 days. In half of the test sessions, we demonstrated action A and in the other half, we demonstrated action B. The action demonstrated (A or B) was semi-randomized, so that the same action would not be demonstrated in more than 2 sessions in a row. We kept the number of sessions and trials limited to ensure that the cat would remain motivated. Due to unforeseen circumstances, on one day, it was not possible to test the cat (the alarm of the pet shop got activated, the cat was frightened and hid behind a closet for the whole test). Therefore, we did not test the cat on that day and, instead, we ran two test sessions in the next test occasion, two days later. Ebisu’s health condition did not allow further testing; therefore, we had to refrain from carrying out more test sessions and from testing her on other actions.

### Data collection and analysis

Video recordings of the cat’s behaviour following the ‘Do it!’ command were used to investigate whether she matched the demonstrated action. Out of the 18 trials, 2 trials were excluded from analysis, one because the cat did not watch the demonstration, and the other because the cat responded by touching the object with both her face and her paw (this happened in the first trial and this trial was excluded from the action matching analysis, but not from the object matching analysis).

We carried out two analyses. Although our aim was testing presence of action matching, we observed that, unexpectedly, in some trials, the cat matched the demonstrated movements but did not perform them on the object (see results). Therefore, we carried out two separate analysis, one for action matching and one for object matching.

To ensure unbiased coding, videos of the cats’ behaviour after the “Do it!” were watched by the coder without knowing what action had been demonstrated. The cat’s behaviour was noted and then compared to the description of the demonstrations to determine action matching. For the action matching analysis, the response of the cat was considered as matching the demonstrated action if the cat performed a movement similar to the one demonstrated by the owner, using a matching body part: when the human demonstrator raised a hand to touch the object, the cat’s response was considered as matching if it raised a front paw either to touch an object or mimicking the movement of raising a hand/paw without touching an object (both coded as “paw action”—action A); when the human demonstrator rubbed her face on the object, the cat’s response was considered as matching if she rubbed her face on something, or if she mimicked the face-rubbing movement without touching any object (both coded as “face action”—action B). If the cat performed any other than the demonstrated action, including action A when action B was demonstrated or vice versa, an action that differed from action A and B, or no action at all, action matching was considered unsuccessful. For the object matching analysis, the response of the cat was considered as matching the object if the cat interacted with any body part with the object touched by the demonstrator.

Statistical analyses were carried out using the R statistical environment (v. 3.4.2, R Development Core Team 2017). First, we calculated whether the cat matched the demonstrations above chance using Binomial tests, setting chance level at 0.5 because there were two actions that were demonstrated. However, we note that this is a conservative analysis, because the cat could potentially perform any behaviour, not only the two demonstrated actions (so that the applied 0.5 chance level is an upper limit).

Then, we followed the analysis of the two-action procedure described by (Akins and Zentall 1996), but adapted it to our experimental design and the single binary response variable we had for each test. In this analysis, we focused on the tests in which the cat performed action A or B (15 trials) and introduced a new variable ('action A') which was coded as ‘1’ if the action performed by the cat was A and ‘0’ if the performed action was B. Action A (binary response variable) was then analysed in binomial Generalized Linear Models (GLM, R package ‘lme4’, Bates et al., 2014) with demonstrated action as an explanatory variable (factor with two levels: A or B). Initial models of action matching included test session and trial, but these variables were kept in the final model only if they had a significant effect (based on AIC values). We used likelihood ratio tests (LRT) to investigate the effects of explanatory variables; we report *χ*^2^ and *P*-values of likelihood ratio tests of models including and excluding the explanatory variable. If action matching was observed, as opposed to other alternative processes, such as stimulus enhancement or goal emulation (Zentall 2006), we expected the demonstrated action to explain whether action A or B was performed by the cat.

For the object matching analysis, we calculated whether the cat interacted with the object more often than what was expected by chance using a Binomial test, considering the two possibilities of whether or not the cat interacted with the object.

## Results

### Action matching

Overall, the cat matched her behaviour to the demonstrated actions in 81.2% trials (13 out of 16 trials, Binomial probability *P* = 0.012). Out of the 9 trials in which a hand movement was demonstrated, the cat responded with a paw movement in 7 trials (77.8%) and out of the 7 trials in which face-rubbing was demonstrated, the cat responded with rubbing her face in 6 trials (85.7%).

The cat performed either action A or B in 15 of 16 trials (i.e. in 93.75% of trials); our statistical analysis confirmed that the demonstrated action explained most of the variations in performed action (GLM of action matching, effect of demonstrated action: *χ*^2^ = 11.193, *df* = 1, *P* < 0.001.

We did not find a significant effect of test session (GLM of action matching, effect of test session: *χ*^2^ = 5.71, *df* = 4 *P* = 0.221) and we also did not find any effect of trial (GLM of action matching, effect of trial: *χ*^2^ = 1.02, *df* = 1, *P* = 0.313); therefore, these variables were excluded from the final model.

### Object matching

Out of the 17 trials analysed for object matching (here we also included the trial in which the cat performed both actions A and B on the box—see above), the cat interacted with the object that was touched by the demonstrator in 11 trials (Binomial probability of interacting *P* = 0.166). In 3 trials in which face-rubbing on the object was demonstrated, the cat responded by rubbing her face on the floor (i.e., on the table’s surface) rather than on the object, and in 1 trial in which the demonstrator raised her hand to touch the object, the cat responded by raising her paw without approaching the object.

For a more detailed description of the cat’s responses, see Table 3.

**Table 3:** Description of the cat’s behaviour in the 18 test trials

Date 12.19	Test session	Trial No	Demo	Cat action	Action Matching
23	1	1	F	E approached the box, raised a paw, touched the box with it and rubbed her face against it	Excluded from analysis
23	1	2	F	“Do it!” not given; E. did not watch the demonstration	Excluded from analysis
23	1	3	F	E. approached the box and rubbed her face against it	1
24	2	4	P	E. approached the box, raised a paw and touch the box with it	1
24	2	5	P	E. approached the box, raised a paw and touch the box with it	1
24	2	6	P	E. approached the box, raised a paw and touch the box with it	1
26	3	7	F	E. rubbed her face on the floor	1
26	3	8	F	E. approached the box and rubbed her face against it	1
26	3	9	F	E. rubbed her face on the floor	1
29	4	10	P	E. raised a paw while not moving her sitting starting position	1
29	4	11	P	E. approached the box, raised a paw and touch the box with it	1
29	4	12	P	E. approached the box, raised a paw and touch the box with it	1
29	5	13	P	E. approached the box, raised a paw and touch the box with it	1
29	5	14	P	E. approached the box and rubbed her face against it	0
29	5	15	P	E. approached the box and rubbed her face against it	0
30	6	16	F	E. approached the box and rubbed her face against it	1
30	6	17	F	E. approached the box and rubbed her face against it	1
30	6	18	F	E. did not move for 30 s., after which the test was stopped	0

The demonstrated actions (“demo”) are indicated as P or F. P refers to the owner raising her hand and touching the box with it; F refers to the owner bending her body down and rubbing her face on the box. “Cat action” refers to the behaviour of the cat (E) following the “Do it!” command. Trials were terminated after 30 s

## Discussion

Our results show the first experimental evidence of the domestic cat’s ability of matching actions to the actions displayed by a heterospecific, human demonstrator in the Do as I Do paradigm. Thereby we provide evidence that the capacity of reproducing actions of a heterospecific model could be considered within the range of cats’ cognitive skills.

Based on the cat’s performance, we argue that she has the ability to map the different body parts and movements of the human demonstrator into her own body parts and movements, at least to some extent. Ebisu’s ability to reproduce the demonstrator’s actions when different actions were shown on the same object allow excluding that behavioural similarity relied only on perceptual factors, such as increased attention to the stimulus. In fact, the cat’s flexibly modified her behaviour based on the different actions that were demonstrated, thereby excluding stimulus enhancement and goal emulation as explanations for the behavioural similarity between demonstrator and observer (Dawson and Foss, 1965; Akins and Zentall 1996; van de Waal et al. 2012).

The two actions chosen as demonstrations were of similar difficulty for the cat and this is confirmed by similar success in reproducing those. One of the two actions—the paw action—was not completely novel for the cat, since she had been trained to touch other objects with her paw. In the case of this action, therefore, the novelty in the test consisted of the object to be touched. However, the face action had not been previously trained, and Ebisu had never been required to perform or imitate this action before the experiment. Her reproduction of the face-rubbing action since the first trial when this action was demonstrated indicates that she was able to generalize the Do as I Do rule to reproduce this action too. This also suggests that cats may have the ability to map the different body parts and movements of the human demonstrator into their own body parts. Face-rubbing is a behaviour that pertains to the natural repertoire of cats (Machado and Genaro 2014; Vitale Shreve and Udell 2017b). However, this action was not included in the Do as I Do training, and Ebisu had never been trained to perform it. Transfer tests of this kind, in which successful performance on one cognitive task is applied to another, ensure that the subjects learned a rule and not a stimulus–response association (Shea and Heyes 2010).

Importantly, in the very first trial when rubbing face on the box was demonstrated (trial 1, Table 3), Ebisu performed both actions: she touched the box with her paw (a body movement that belonged to her training repertoire) and she also rubbed her face on the box. Although this trial was excluded from the action matching analysis due to its ambiguity, we note that the cat performed the demonstrated action after the very first time seeing it and this shows that the cat was already able to use the demonstration as a sample against which to match her behaviour at the start of the experiment. The performance of the cat can be explained by imitation (Whiten and Ham 1992) or, alternatively, by response facilitation (Byrne 1994).

Unexpectedly, Ebisu did not always approach the object used by her owner during the demonstrations and in 4 trials she performed the demonstrated action “on nothing” or on the floor (so-called “vacuum actions”, Huber et al. 2009). This happened in three face action trials and in one paw action trials, suggesting that it was not an action-specific response. Moreover, the cat did not approach the object (and location) where the demonstration was performed more likely than chance level. This may simply be due to fatigue and reduced motivation related to the compromised health of the cat (i.e., it may be due to tiredness or low motivation, making it more likely that the subject would save energy and not move from her starting position).

Ebisu’s health condition did not allow further testing; therefore, some caution should be taken before generalizing the results to other actions, not tested in the present study. However, the results obtained by combining the Do as I Do method and the two-action procedure allow us to exclude that the cat’s performance relied on other processes, such as stimulus enhancement or goal emulation. These findings provide evidence that the cat was able to successfully learn to reproduce human-demonstrated actions with the Do as I Do method. Cats, similar to dogs (e.g. Fugazza and Miklósi 2014), might be able to map the different body parts and movements of the human demonstrator into their own body parts, at least with regard to the tested actions. Ebisu’s motivation for food and training activities made it possible to successfully train her with the Do as I Do method. Our experience about the time investment and difficulty of training cats prevented us from testing other subjects, therefore, the extent to which we can generalize these results to the cat population in general takes further investigation. We suggest that cats possess the cognitive skill to reproduce the actions of conspecific and—if properly socialized—also heterospecific models. Therefore, we think that these results could be replicated, provided that the subjects can be motivated enough by food, toys/play or social reward, to collaborate with a human trainer.

## Electronic supplementary material

Below is the link to the electronic supplementary material.

Supplementary file1 (MP4 457749 kb)

## Data Availability

Data files are available upon request.
